# The Application Effect of Endoscopic Thyroidectomy via the Gasless Unilateral Axillary Approach in Thyroid Cancer and Its Impact on Postoperative Stress Response

**DOI:** 10.3390/curroncol32050252

**Published:** 2025-04-26

**Authors:** Jinliang Jia, Jihua Han, Rui Pang, Wen Bi, Bo Liu, Ruinan Sheng, Lingyu Kong

**Affiliations:** Department of Head and Neck Surgery, Harbin Medical University Cancer Hospital, Harbin 150081, China; jiajinliang792@outlook.com (J.J.); hanjihua177@outlook.com (J.H.); pangrui845@outlook.com (R.P.); biwen4388@outlook.com (W.B.); liubo2691@outlook.com (B.L.); shengruinan330@outlook.com (R.S.)

**Keywords:** thyroid cancer, axillary approach, gasless endoscopic thyroidectomy, parathyroid function, stress response

## Abstract

Objective: This study aims to evaluate the application effect of endoscopic thyroidectomy via the gasless unilateral axillary (GUA) approach in thyroid cancer and its impact on the postoperative stress response. Methods: Ninety-four thyroid cancer patients were enrolleod and assigned into the open group (underwent conventional-open-anterior-cervical-approach thyroidectomy) and the endoscopic group (underwent GUA endoscopic thyroidectomy) (n = 47). Perioperative indicators between the two groups were compared. Thyroid function parameters [total triiodothyronine (TT3), total thyroxine (TT4), free triiodothyronine (FT3), free thyroxine (FT4), and thyroid-stimulating hormone (TSH)] were measured preoperatively and on postoperative day 2. Inflammatory markers [interleukin-6 (IL-6) and tumor necrosis factor-α (TNF-α)] and stress-related hormones [norepinephrine (NE) and cortisol (Cor)] were evaluated preoperatively and on postoperative day 1. The aesthetic appearance of the incision was evaluated at 1 and 3 months postoperatively using the Vancouver Scar Scale (VSS). Postoperative complications were also compared between the two groups. Results: The endoscopic group exhibited less intraoperative blood loss, reduced postoperative drainage, a lower pain degree on 1 day postoperatively, a shorter hospitalization time, and a longer surgical time versus the open group (*p* < 0.05). The serum levels of TT3, TT4, FT3, and FT4 were lower, while the TSH levels were higher in both groups on postoperative day 2 compared to preoperative values. Additionally, the serum levels of IL-6, TNF-α, NE, and Cor increased on day 1 postoperatively, with the endoscopic group showing lower levels of these markers compared to the open group (*p* < 0.05). The VSS scores at 1 and 3 months after surgery were lower in the endoscopic group compared to the open group, indicating better cosmetic outcomes (*p* < 0.05). The incidence of postoperative complications was comparable between the endoscopic and open groups (*p* > 0.05). Conclusions: Endoscopic thyroidectomy by a GUA offers notable advantages over the conventional-open-anterior-cervical-approach thyroidectomy, including reduced intraoperative blood loss, less postoperative drainage, and a lower postoperative stress response. This approach also results in improved cosmetic outcomes, making it a promising alternative for thyroid cancer surgery.

## 1. Introduction

Thyroid cancer is a heterogeneous malignancy that originates in the thyroid gland, making it the predominant endocrine malignancy around the world [1]. Thyroid cancer’s incidence continues to rise, placing significant psychological and economic burdens on both individuals and society [2]. Despite advances in treatment, thyroid cancer remains challenging [3], particularly undifferentiated tumors, which often show a limited response to standard therapies [4]. This highlights the urgent need for advanced diagnosis and management strategies [5].

Conventional open thyroidectomy, which remains the standard surgical approach for thyroid cancer, often leaves visible scars on the neck, affecting patients’ quality of life [6]. Endoscopic thyroidectomy, a relatively novel technique, has gained attention in the treatment of differentiated thyroid cancer. Various surgical approaches have been developed, offering potential advantages over traditional surgery, Specifically, scarless endoscopic thyroidectomy has been reported to yield superior cosmetic outcomes and lower incidences of hypesthesia, paresthesia, and psychological distress related to visible scarring [7]. Endoscopic thyroidectomy for papillary thyroid carcinoma, in particular, is associated with mild postoperative stress responses, excellent cosmetic effects, and fewer complications [8]. The exposure of the recurrent laryngeal nerve in thyroid surgery is essential [9]. Endoscopic thyroidectomy is considered an effective and safe option for patients with thyroid cancer, offering various approaches tailored to patient needs. Among these, the gasless unilateral axillary (GUA) approach has emerged as a promising technique, providing enhanced cosmetic satisfaction for patients [10]. GUA endoscopic thyroidectomy has gained widespread use globally [11], and is regarded as superior to traditional unilateral incisions for total thyroidectomy, offering excellent cosmetic results [12]. In addition, GUA endoscopic thyroidectomy is associated with several advantages, including ease of manipulation, clear visualization, a short learning curve, concealed incisions, no visible neck scars, and less postoperative swallowing discomfort [13]. Inspired by these findings, our study aimed to evaluable the application effect of an endoscopic thyroidectomy via the GUA approach in thyroid cancer and its impact on the postoperative stress response.

## 2. Materials and Methods

### 2.1. Ethics Statement

The research was approved by the Ethics Committee of Harbin Medical University Cancer Hospital (approval number: 2021082), and all participants provided written informed content.

### 2.2. General Data

Ninety-four thyroid cancer patients who were admitted to the Head–Neck Surgery Department of Harbin Medical University Cancer Hospital between March 2022 and April 2024 were collected in the study. These patients were grouped into two groups according to different surgical approaches, the open group and the endoscopic group, with 47 cases in each group.

### 2.3. Inclusion Criteria

① Patients who met the diagnostic criteria for thyroid cancer [14], confirmed by vocal cord examination, ultrasound, parathyroid function test, biopsy, and enhanced CT of the neck. ② Patients who were suitable candidates for surgery, i.e., in good physical condition and able to tolerate general anaesthesia. The tumor was located in the unilateral thyroid lobe, with a diameter of ≤4 cm, confined to the thyroid gland without invasion of adjacent structures (e.g., trachea, recurrent laryngeal nerve, or esophagus). Thyroid function was normal or mildly abnormal, with no evidence of distant metastasis or lymph node metastasis, or only limited central lymph node metastasis. ③ Patients aged 18–60 years old. ④ Patients undergoing thyroid surgery for the first time. ⑤ Patients with normal cognition abilities, who are capable of listening, speaking, reading, and writing. ⑥ Patients who were able to cooperate with the treatment and research, and who had complete clinical data.

### 2.4. Exclusion Criteria

① Patients with a history of neck radiotherapy or prior neck surgery. ② Patients with drug or alcohol dependence. ③ Patients with other concurrent tumors. ④ Patients with thyroid diseases such as thyroiditis and hyperthyroidism. ⑤ Patients with organic diseases affecting critical organs (e.g., heart, liver, and kidneys). ⑥ Patients with immune system dysfunction. ⑦ Patients with coagulation disorders or endocrine disorders. ⑧ Patients with infectious diseases or acute or chronic systemic infections. ⑨ Patients who withdrew from the study or transferred to another hospital during the study period for any reason.

### 2.5. Methods

All patients underwent ipsilateral thyroid lobectomy and isthmectomy combined with ipsilateral central lymph node dissection. Based on this, the two groups were treated with different surgical approaches.

(1) The open group (conventional-open-anterior-cervical-approach thyroidectomy): the patients underwent general anaesthesia with tracheal intubation, and were placed in a supine position with their upper limbs abducted. Routine disinfection and draping were conducted. An arc-shaped incision was made along the skin creases at the level of the clavicle. The flap was carefully dissected from top to bottom, and the linea alba cervicalis was incised to fully expose the thyroid gland. The upper, middle, and lower thyroid blood vessels were managed, and the recurrent laryngeal nerves as well as any suspicious parathyroid tissue were identified and protected. The thyroid lobes and isthmus were resected, and lymph nodes in the central region were excised. Following the resection, the area was irrigated, hemostasis was achieved, and layer-by-layer suturing was performed, with drainage placement to complete the procedure.
(2) The endoscopic group (GUA endoscopic thyroidectomy): the patients received general anaesthesia with tracheal intubation and were positioned supine with their upper limbs abducted. Routine disinfection and draping were conducted. A 4 cm incision was made in the axillary fold on the affected side, extending toward the sternum along the space between the sternal and clavicular heads of the sternocleidomastoid muscle. The sternal head of the muscle was elevated using a retractor, and the outer edge of the anterior cervical banded muscle was separated to expose the thyroid lobe. A suspension system was utilized to create the surgical cavity. The upper and lower poles of the thyroid arteries and veins were cauterized with an ultrasound knife, and the upper parathyroid glands were retained by the decapitation method. Special care was taken to protect the recurrent laryngeal nerves throughout the procedure. After the thyroid lobes and isthmus were removed and central lymph node dissection was completed, the surgical area was irrigated, hemostasis was achieved, and layer-by-layer suturing was performed, followed by drainage placement.

### 2.6. Observation Indicators

(1) Perioperative indicators are as follows: intraoperative blood loss, postoperative drainage, number of lymph node dissection in the central region, pain degree 1 day postoperatively (assessed using the visual analogue scale (VAS), ranging from 0 for no pain to 10 for severe pain) [15]), surgical time, and hospitalization time.
(2) Thyroid function: Before and 2 days after surgery, 3 mL of fasting elbow vein blood was collected from patients in both groups. The blood was centrifuged at 3000 r/min for 10 min with a rotor radius of 5 cm, and the serum was separated. The levels of total triiodothyronine (TT3), total tetraiodothyronine (TT4), free triiodothyronine (FT3), free tetraiodothyronine (FT4), and thyroid-stimulating hormone (TSH) were tested using a fully automated biochemical analyzer (iChem 520; Shenzhen Icubio Biomedical Technology Co., Ltd., Shenzhen, China).
(3) Inflammatory factors: Before surgery and 1 day after surgery, 3 mL of fasting elbow venous blood was collected and centrifuged as described above. Serum levels of interleukin-6 (IL-6) and tumor necrosis factor-α (TNF-α) were assessed by enzyme-linked immunosorbent assay (ELISA) using kits purchased from eBioscience (San Diego, CA, USA).
(4) Stress response indicators: Prior to surgery and 1 day after surgery, 3 mL of fasting elbow vein blood was collected and processed as described above. The serum levels of norepinephrine (NE) and cortisol (Cor) were measured by ELISA, with kits from eBioscience (San Diego, CA, USA).
(5) Incision aesthetics: The incision aesthetics was assessed by the Vancouver Scar Scale (VSS) at 1 and 3 months postoperatively. The VSS assesses 4 aspects: colour, vascular distribution, thickness, and softness, with a total score of 0–15 points. Lower scores correspond to better aesthetic outcomes.
(6) Postoperative complications: Patients were followed up for 3 months after surgery. The complications assessed included temporary recurrent laryngeal nerve paralysis, parathyroid gland injury, incision infection, and hypocalcaemia.

### 2.7. Statistics

Statistical analysis was performed using SPSS 26.0 software. Categorical data were described by [n (%)] and χ^2^ test was applied. Normally distributed continuous data were depicted as $\bar{x}$ ± s and independent- or paired-sample *t*-tests were used for comparisons. Skewed data were represented as median (P25 and P75), and the Mann–Whitney U-test was applied. *p* < 0.05 was considered as statistically significant difference.

## 3. Results

### 3.1. Baseline Characteristic

The general characteristics of patients, including gender, age, maximum tumor diameter, tumor location, number of tumors, pathological type, and TNM staging were collected for statistical analysis. No significant differences were found between the open and endoscopic groups (*p* > 0.05) (Table 1).

### 3.2. Perioperative Indicators

The endoscopic group exhibited less intraoperative blood loss, reduced postoperative drainage, a lower pain degree 1 day postoperatively, a shorter hospitalization time, and a longer surgical time versus the open group (*p* < 0.05). There were no significant differences between the two groups regarding the number of lymph node dissection in the central region (*p* > 0.05) (Table 2).

### 3.3. Thyroid Function

Preoperative serum TT3, TT4, FT3, FT4, and TSH levels were comparable between the two groups (*p* > 0.05). Two days postoperatively, the serum TT3, TT4, FT3, and FT4 levels decreased in both groups, while the serum TSH levels increased (*p* < 0.05). However, no significant differences were observed between the two groups for these parameters (*p* > 0.05) (Table 3).

### 3.4. Inflammatory Factors

No significant difference was noted in preoperative serum IL-6 and TNF-α levels between the two groups (*p* > 0.05). One day postoperatively, both groups exhibited elevated serum IL-6 and TNF-α levels, and these levels were lower in the endoscopic group versus in the open group (*p* < 0.05) (Table 4).

### 3.5. Stress Response

No significant difference was noted in the preoperative serum NE and Cor contents between the two groups (*p* > 0.05). One day after surgery, both groups showed elevated serum NE and Cor, and the contents in the endoscopic group were lower versus the open group (*p* < 0.05) (Table 5).

### 3.6. Incision Aesthetics

The VSS scores at 1 and 3 months after surgery in the endoscopic group were lower versus those of the open group, indicating better aesthetic outcomes in the endoscopic group (*p* < 0.05) (Table 6).

### 3.7. Postoperative Complications

The incidence of postoperative complications was 6.38% in the endoscopic group and 12.77% in the open group. There was no significant difference between the two groups in the incidence of complications (*p* > 0.05) (Table 7).

## 4. Discussion

The incidence of thyroid cancer, the most prevalent endocrine cancer [16], is increasing globally. Thyroid cancer’s etiology is complicated, involving the multiple interplay of factors that interact in multifaceted ways [17]. Therefore, this paper was aimed at exploring the application effect of an endoscopic thyroidectomy via the GUA approach in thyroid cancer and its impact on the postoperative stress response.

As previously reported, the prevalence of low-risk differentiated thyroid cancer has surged, likely due to advancements in diagnostic imaging technologies. Additionally, remote-access endoscopic thyroidectomy, which eliminates visible neck scars, has gained popularity [18]. GUA thyroidectomy has made significant technological strides and is now being increasingly utilized [19]. In our study, we compared perioperative indicators between the endoscopic group (GUA endoscopic thyroidectomy) and the open group (conventional open thyroidectomy) and it was found that the endoscopic group exhibited less intraoperative blood loss, reduced postoperative drainage, a lower pain degree 1 day postoperatively, a shorter hospitalization time, and a longer surgical time versus the open group. Importantly, there was no significant difference in the number of central lymph nodes dissected between the two groups. These findings align with previous studies, which also noted that the number of central lymph nodes dissected in GUA endoscopic thyroidectomy was comparable to that in conventional open thyroidectomy, although the operation time for the GUA endoscopic thyroidectomy was longer [20].

Next, we compared the thyroid function between the two groups and found that the serum TT3, TT4, FT3, and FT4 contents in the two groups two days after surgery were lower versus those before surgery, while the serum TSH levels were higher. When the body is subjected to trauma, the intensity of the stress response correlates positively with the degree of surgical trauma. Systemic reactions include the activation of the monocyte–macrophage system, the release of cytokines, and the resultant pathophysiological changes. Laboratory indicators such as TNF-α and IL-6 concentrations are elevated during this process. In this study, we observed postoperative elevations in TNF-α and IL-6 levels in both groups. Crippa et al. [21] suggested that monitoring IL-6 and TNF-α concentrations could evaluate the extent of surgical trauma. Our findings indicated that postoperative TNF-α and IL-6 levels were lower in the endoscopic group than in the open group, suggesting that endoscopic surgery is associated with a smaller stress response. Cor and NE are key indicators of the stress response. Cor, primarily secreted by the adrenal cortex, is regulated by the hypothalamic corticotropin-releasing hormone and pituitary adrenocorticotropic hormone, and is a sensitive marker of the stress response. Sympathetic–adrenal medullary system excitation is reflected in significantly elevated serum NE levels. Surgery, as a traumatic intervention, induces a stress response that results in increased serum Cor and NE levels. In this study, we observed elevated serum Cor and NE levels postoperatively in both groups. However, the postoperative Cor and NE levels were lower in the endoscopic group than in the open group, indicating that endoscopic thyroid surgery induces a smaller stress response in patients.

Long-term postoperative cosmetics following GUA endoscopic thyroidectomy are superior to those of conventional open thyroidectomy. Moreover, the endoscopic group demonstrated higher cosmetic satisfaction and lower scar awareness scores [22]. GUA endoscopic thyroid surgery is considered a safe treatment option with a lower incidence of anterior cervical discomfort during swallowing [23]. No significant difference in the occurrence of surgical complication has been reported between conventional open thyroidectomy and endoscopic thyroidectomy [10]. In our research, incision aesthetics was assessed using the VSS and it was found that the VSS scores at 1 and 3 months after surgery were lower in the endoscopic group versus the open group. Furthermore, the incidence of postoperative complications did not differ significantly between the two groups.

Our study is on the basis of limited clinical data, and, therefore, our study results are only representative for a small group of patients with thyroid cancer. Another limitation of this study is the lack of cases involving severe stress-related complications, which limits the ability to fully assess the clinical implications of the stress response in this context. While the absence of severe complications suggests that the surgical approach may minimize stress-induced risks, it also restricts the generalizability of these findings to cases with higher stress levels or more complex patient profiles. Future studies with larger sample sizes and diverse patient populations, including those with severe stress responses, are needed to comprehensively evaluate the role and clinical significance of stress responses in similar surgical procedures.

In conclusion, this research unravels the idea that, compared to the conventional-open-anterior-cervical-approach thyroidectomy, the application of endoscopic thyroidectomy by a GUA in thyroid cancer patients demonstrates more significant advantages, including reduced intraoperative blood loss and postoperative drainage volume, as well as effectively mitigating the traumatic stress response in patients. These benefits suggest that the GUA approach is a safer and less invasive surgical option, potentially improving postoperative recovery and overall patient outcomes. Its ability to achieve comparable surgical efficacy while offering reduced surgical trauma and better cosmetic results underscores its value in modern thyroid cancer management.

## Figures and Tables

**Table 1:** General data between the two groups [n (%)].

Indicators	The Endoscopic Group (n = 47)	The Open Group (n = 47)	*χ* ^2^	*p*
Gender			0.237	0.626
Male	12 (25.53)	10 (21.28)	-	-
Female	35 (74.47)	37 (78.72)	-	-
Age			0.783	0.376
18–40 years old	17 (36.17)	13 (27.66)	-	-
41–60 years old	30 (63.83)	34 (72.34)	-	-
Maximum tumor Diameter			1.659	0.198
≤10 mm	14 (29.79)	20 (42.55)	-	-
>10 mm	33 (70.21)	27 (57.45)	-	-
Tumor location			0.170	0.680
Left lobe	24 (51.06)	22 (46.81)	-	-
Right lobe	23 (48.94)	25 (53.19)	-	-
Number of tumors			0.384	0.536
Single	26 (55.32)	23 (48.94)	-	-
Multiple	21 (44.68)	24 (51.06)	-	-
Pathological type			0.714	0.398
Papillary carcinoma	41 (87.23)	38 (80.85)	-	-
Follicular carcinomas	6 (12.77)	9 (19.15)	-	-
TNM staging			0.389	0.533
I stage	19 (40.43)	22 (46.81)	-	-
II stage	28 (59.57)	25 (53.19)	-	-

**Table 2:** Perioperative indicators between the two groups.

Indicators	The Endoscopic Group (n = 47)	The Open Group (n = 47)	*Z*/*t*	*p*
Intraoperative blood loss (mL)	20.00 (17.00, 22.00)	31.00 (29.00, 34.00)	−8.320	<0.001
Postoperative drainage (mL)	77.98 ± 9.93	127.21 ± 9.40	−24.676	<0.001
Number of lymph node dissection in the central region (numbers)	3.00 (3.00, 3.00)	3.00 (2.00, 3.00)	−0.941	0.347
Pain degree 1 day postoperatively (points)	4.00 (3.00, 4.00)	6.00 (5.00, 6.00)	−7.839	<0.001
Surgical time (min)	122.98 ± 8.18	81.98 ± 9.93	21.848	<0.001
Hospitalization time (d)	5.00 (5.00, 6.00)	7.00 (6.00, 7.00)	−5.532	<0.001

**Table 3:** Thyroid function between the two groups.

Time	The Endoscopic Group (n = 47)	The Open Group (n = 47)	*t*	*p*
Preoperatively				
TT3 (pg/mL)	1.84 ± 0.28	1.86 ± 0.30	−0.346	0.730
TT4 (pg/mL)	104.72 ± 8.44	103.84 ± 8.63	0.500	0.619
FT3 (pg/L)	4.67 ± 0.45	4.72 ± 0.47	−0.536	0.594
FT4 (pg/L)	16.28 ± 1.37	16.15 ± 1.47	0.442	0.659
TSH (mU/L)	2.50 ± 0.30	2.48 ± 0.27	0.350	0.727
2 days postoperatively				
TT3 (pg/mL)	1.12 ± 0.30 ^a^	1.03 ± 0.20 ^a^	1.772	0.080
TT4 (pg/mL)	75.39 ± 5.20 ^a^	73.96 ± 5.48 ^a^	1.299	0.197
FT3 (pg/L)	2.70 ± 0.33 ^a^	2.64 ± 0.28 ^a^	1.005	0.317
FT4 (pg/L)	11.74 ± 1.40 ^a^	11.41 ± 1.00 ^a^	1.314	0.192
TSH (mU/L)	4.32 ± 0.38 ^a^	4.21 ± 0.44 ^a^	1.306	0.195

Note: ^a^ *p* < 0.05 vs. the same group preoperatively.

**Table 4:** Inflammatory factors between the two groups.

Time	The Endoscopic Group (n = 47)	The Open Group (n = 47)	*t*	*p*
Preoperatively				
IL-6 (pg/mL)	0.20 ± 0.06	0.22 ± 0.09	1.268	0.208
TNF-α (pg/mL)	0.66 ± 0.15	0.70 ± 0.29	1.050	0.297
1 day postoperatively				
IL-6 (pg/mL)	2.70 ± 0.72 ^a^	2.98 ± 1.04 ^a^	3.594	0.001
TNF-α (pg/mL)	5.02 ± 1.57 ^a^	6.24 ± 1.69 ^a^	3.626	0.001

Note: ^a^ *p* < 0.05 vs. the same group preoperatively. IL-6 normal range: 0–7 pg/mL; TNF-α normal range: 0–8.1 pg/mL.

**Table 5:** Stress response between the two groups.

Time	The Endoscopic Group (n = 47)	The Open Group (n = 47)	*t*	*p*
Preoperatively				
Cor (mmol/L)	118.86 ± 9.76	117.71 ± 11.18	0.533	0.596
NE (pg/mL)	515.15 ± 24.46	516.94 ± 33.16	−0.297	0.767
1 day postoperatively				
Cor (mmol/L)	123.21 ± 6.44 ^a^	138.84 ± 7.80 ^a^	−10.594	<0.001
NE (pg/mL)	527.62 ± 34.44 ^a^	656.79 ± 34.33 ^a^	−18.210	<0.001

Note: ^a^ *p* < 0.05 vs. the same group preoperatively.

**Table 6:** Incision aesthetics between the two groups (points).

Time	The Endoscopic Group (n = 47)	The Open Group (n = 47)	*Z*	*p*
VSS at 1 month postoperatively	5.00 (4.00, 6.00)	7.00 (6.00, 7.00)	−6.820	<0.001
VSS at 3 months postoperatively	4.00 (3.00, 4.00)	5.00 (4.00, 6.00)	−5.774	<0.001

**Table 7:** Postoperative complications between the two groups [n (%)].

Complications	The Endoscopic Group (n = 47)	The Open Group (n = 47)	*χ* ^2^	*p*
Temporary recurrent laryngeal nerve paralysis	2 (4.26)	1 (2.13)	-	-
Parathyroid gland injury	1 (2.13)	3 (6.38)	-	-
Incision infection	0 (0.00)	1 (2.13)	-	-
Hypocalcaemia	0 (0.00)	1 (2.13)	-	-
Total	3 (6.38)	6 (12.77)	1.106	0.486

## Data Availability

The data that support the findings of this study are not publicly available due to their containing information that could compromise the privacy of research participants but are available from the corresponding author upon reasonable request.
